# Telomere-to-telomere gapless genome assembly of the Chinese sea bass (*Lateolabrax maculatus*)

**DOI:** 10.1038/s41597-024-02988-9

**Published:** 2024-02-07

**Authors:** Zhilong Sun, Shuo Li, Yuyan Liu, Weijing Li, Kaiqiang Liu, Xuebin Cao, Jiliang Lin, Hongyan Wang, Qian Wang, Changwei Shao

**Affiliations:** 1 College of Marine Technology and Environment, Dalian Ocean University, Dalian, 116023 China; 2 National Key Laboratory of Mariculture Biobreeding and Sustainable Goods, Yellow Sea Fisheries Research Institute, Chinese Academy of Fishery Sciences, Qingdao, Shandong 266071 China; 3 Yantai Jinghai Marine Fisheries Co., Ltd, Yantai, 264000 China

**Keywords:** Genomics, Genome informatics

## Abstract

Chinese sea bass (*Lateolabrax maculatus*) is a highly sought-after commercial seafood species in Asian regions due to its excellent nutritional value. With the rapid advancement of bioinformatics, higher standards for genome analysis compared to previously published reference genomes are now necessary. This study presents a gapless assembly of the Chinese sea bass genome, which has a length of 632.75 Mb. The sequences were assembled onto 24 chromosomes with a coverage of over 99% (626.61 Mb), and telomeres were detected on 34 chromosome ends. Analysis using Merqury indicated a high level of accuracy, with an average consensus quality value of 54.25. The ONT ultralong and PacBio HiFi data were aligned with the assembly using minimap2, resulting in a mapping rate of 99.9%. The study also identified repeating elements in 20.90% (132.25 Mb) of the genome and inferred 22,014 protein-coding genes. These results establish meaningful groundwork for exploring the evolution of the Chinese sea bass genome and advancing molecular breeding techniques.

## Background & Summary

**Fig. 1:**
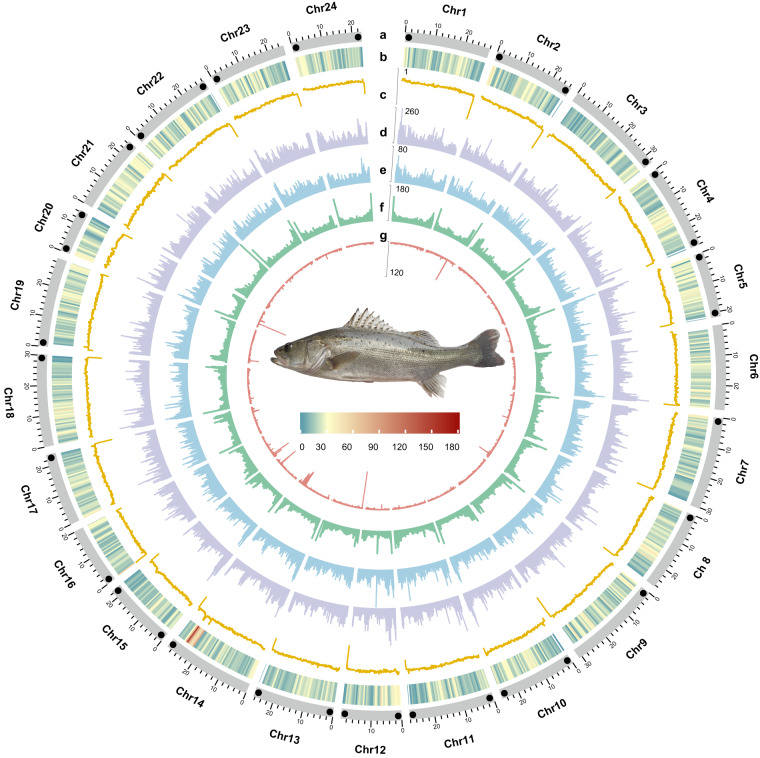
Genomic landscape of the Chinese sea bass. The rings, from the outermost to the innermost layer, represent the chromosomes of the *L. maculatus* genome (**a**), gene density (**b**), GC density (**c**), DNA transposons (**d**), LTRs (**e**), LINEs (**f**), and SINEs (**g**). The identified telomere ends are represented by black dots in (**a**). The analysis of (**a**) was conducted using 500-kb genomic windows, while (**b**–**g**) were analysed using 50-kb sliding windows.

The Chinese sea bass (*Lateolabrax maculatus*) (Fig. 1), a member of the Moronidae family in the Perciformes order, displays a distinctive feature of multiple prominent black dots on its lateral body region^1^. Recently, it has been distinguished as a new species with obvious morphological and genetic differences from the Japanese sea bass (*Lateolabrax japonicus*)^2^. Compared to *L. japonicus*, *L. maculatus* has a wider ecological range and is found along the coast and estuaries of China, Japan, and the Korean Peninsula^1^. The Chinese sea bass shows excellent adaptability to a wide range of temperatures and salinity environments and possesses a delicate taste and high nutritional value^1,3,4^. Therefore, it has been extensively cultivated in freshwater ponds and seawater net cages in China^5^. In 2021, the yearly production of Chinese sea bass in China reached 199,106 tons, which accounted for 10.79% of the aggregate aquafarming output of marine fish. Consequently, the Chinese sea bass is regarded as a much sought-after marine economic fish in China^6^.

Recently, extensive molecular genetics research has been conducted on the Chinese sea bass, and the genomes of Chinese sea bass from both the Bohai Gulf and subtropical regions have been assembled^7,8^. Besides, numerous transcriptomic databases have been generated, and extensive research on functional genes has been conducted by researchers^9^. However, with advancements in genome sequencing procedures and DNA assembly methodologies, seamless telomere-to-telomere (T2T) genome assembly has now become a reality, enabling the identification of almost the entire genome. Recently, there has been a surge in deciphering seamless genomes for several species, such as *Arabidopsis thaliana*, *Homo sapiens*, *Citrullus lanatus*, *Clarias gariepinus*, *Musa acuminata*, *Oryza sativa*, and *Fragaria vesca*^10–16^. However, assembly of the *L. maculatus* genome at an equivalent level has not yet been reported.

To this end, we integrated Pacific Biosciences (PacBio) HiFi sequencing, Oxford Nanopore Technologies (ONT) ultralong sequencing, and Hi-C technology to assemble a high-quality T2T genome of *L. maculatus*. Our assembly significantly improves upon the two previously published genome assemblies, as it is nearly complete without any gaps (Fig. 1). This not only facilitates population genetic research and evolutionary analysis of the Chinese sea bass but also provides important resources for optimizing genetic breeding.

## Methods

### Sample collection and sequencing

Mature male Chinese sea bass were captured from the Yantai Jinghai Marine Fisheries Co., Ltd, Yantai Shandong, China. High molecular weight genomic DNA (gDNA) was isolated from muscle tissue using a standard sodium dodecyl sulfate (SDS) extraction method for ONT ultralong sequencing. For PacBio HiFi sequencing, a Blood & Cell Culture DNA Kit (Qiagen 13323) was utilized to extract the gDNA. Three methods were used for DNA quality and quantification testing, including (i) a NanoDrop 2000 spectrophotometer (Thermo Fisher Scientific, USA), (ii) gel electrophoresis, and (iii) a Qubit fluorometer (Invitrogen, USA). Total DNA was purified by AMPure PB beads (PacBio 100-265-900, USA). High-quality gDNA was prepared for the next step of library construction.

The PacBio HiFi sequencing technique included the construction of a standard SMRTbell library using the SMRTbell Express Template Prep Kit 2.0, following the prescribed guidelines from the manufacturer. Subsequently, the SMRTbell libraries underwent sequencing using a PacBio Sequel II system (Pacific Biosciences, CA, USA). For ONT ultralong sequencing, a library was produced with the Oxford Nanopore SQK-ULK001 kit following the instructions provided by the manufacturer and then sequenced on a PromethION flow cell. As a result, 73.83 Gb (117×) of PacBio HiFi read data and 62.58 Gb (99×) of ONT ultralong read data were obtained (Table 1).

**Table 1:** Statistics of the sequencing data.

Library type	Platform	Tissue	Data size (Gb)	Average depth (×)	Average Length (bp)
ONT ultra-long	PromethION	Muscle	62.58	99	97,395
PacBio SMRT	Pacbio Sequel II	Muscle	73.83	117	16,135
Hi-C	Illumina Novaseq 6000	Blood	92.61	146	150

The Hi-C library was produced using a blood sample from the same Chinese sea bass used for gDNA sequencing. Library construction involved the following steps^17,18^: initial crosslinking of cells using formaldehyde, DNA digestion, end filling and biotin labelling, ligation of the generated blunt-end fragments, purification, and random shearing of DNA into 300–500 bp fragments. After a quality control test of the libraries using Qubit 2.0 (Invitrogen, USA), an Agilent 2100 instrument (Agilent Technologies, CA, USA), and q-PCR, 150 bp PE sequencing of the Hi-C library was implemented on the Illumina NovaSeq. 6000 platform. In total, 92.61 Gb (146×) of Hi-C read data was obtained (Table 1).

### Genome assembly and telomere identification

**Fig. 2:**
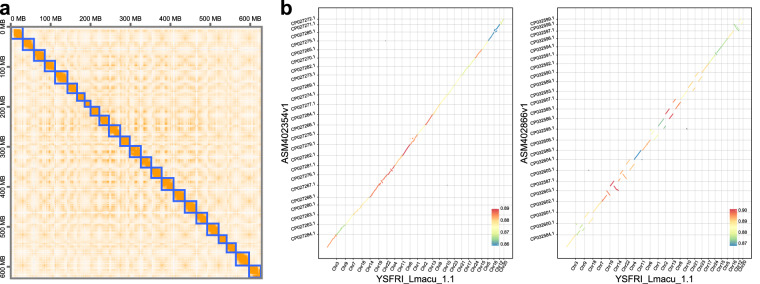
Overview of the genome-wide Hi-C heatmap and collinearity diagram comparing the old and new versions of the genome assembly. (**a**) The Hi-C heatmap illustrates the interaction frequencies among various chromosomes in Chinese sea bass. Chromosomes are represented by blue squares. (**b**) Dot plots illustrate the collinear relationship between the *L. maculatus* assembly and its two previously published assemblies.

With the ultralong ONT, PacBio HiFi, and Hi-C sequencing data described above, the contigs were assembled utilizing the initial values of Hifiasm^19^ (v0.19.5). We obtained a gapless-level genome assembly of *L. maculatus* (YSFRI_Lmacu_1.1), where the genome length was approximately 632.75 Mb and N50 was 27.95 Mb (Table 2). The 3D-DNA pipeline and Juicer-box^20^ (v1.91) were utilized to examine and visualize the interaction frequencies among different chromosomes (Fig. 2a). Both karyotype analysis and the published genome assembly of ASM402354v1 indicate that the species has a total of 24 chromosomes^8,21^. Subsequently, we employed minimap2^22^ (v2.17) to compare the *L. maculatus* genome with the two published genomes. Our assembly appears to be significantly more complete than the current reference genome (ASM402354v1), and it exhibits a distinct mount order in comparison to the other assembly (ASM402866v1) (Fig. 2b). To assess the assembled telomere sequences in the Chinese sea bass genome, we utilized the Telomere Identification toolkit (v0.2.31) (https://github.com/tolkit/telomeric-identifier) to identify occurrences of a 6 bp motif (TTAGGG) within the genome sequence. A total of 34 telomeres were identified, and telomeres were detected on both ends of 11 chromosomes (Fig. 1 and Table 3).

**Table 2:** Assembly statistics of Chinese sea bass.

Assembly	ASM402354v1	ASM402866v1	YSFRI_Lmacu_1.1
Total length	668.45 Mb	597.39 Mb	632.75 Mb
Chromosome length	519.24 Mb	586.03 Mb	626.61 Mb
Chromosome length percentage	77.68%	98.10%	99.03%
Scaffold number	24	24	—
Contig number	22,801	5,016	109
Scaffold N50	1.04 Mb	2.79 Mb	—
Contig N50	31Kb	182 kb	27.95 Mb
BUSCO	86.8%	97.03%	97.9%
Repetitive sequence	138.82 Mb	105.50 Mb	132.25 Mb
Repetitive sequence percentage	20.77%	17.66%	20.90%
Gene prediction	22, 015	23,657	22,014

**Table 3:** Assembly statistics of chromosomes.

Name	Length (bp)	Telomere Number
Chr 1	28,002,630	1
Chr 2	26,705,167	2
Chr 3	32,929,622	1
Chr 4	28,273,811	2
Chr 5	21,653,359	2
Chr 6	27,954,791	0
Chr 7	30,415,611	1
Chr 8	26,556,538	1
Chr 9	31,002,065	1
Chr 10	25,675,649	2
Chr 11	28,162,714	2
Chr 12	19,902,208	2
Chr 13	26,623,924	2
Chr 14	29,626,110	1
Chr 15	22,759,512	2
Chr 16	20,185,356	1
Chr 17	23,914,702	1
Chr 18	30,329,235	1
Chr 19	29,283,490	1
Chr 20	14,559,803	2
Chr 21	24,830,981	1
Chr 22	28,616,760	2
Chr 23	25,526,257	1
Chr 24	23,123,726	2
85 unplaced contigs	6,136,962	—

### Repetitive sequence annotation

We utilized a combined approach involving *de novo* explorations and homologous alignments for the annotation of repeat elements. Homologue prediction was performed using RepeatMasker^23^ (v4.0.6) and RepeatProteinMask^24^ (v4.0.6) based on the Repbase library^25^ (v202101). Tandem Repeats Finder^26^ (v4.07) was utilized specifically for the detection of tandem repeats. RepeatModeler^24^ (v1.0.8) and LTR-Finder^27^ (v1.06) were employed for *de novo* prediction of repeat elements. The resultant predictions were merged to create a library utilized by RepeatMasker for the identification of repeat elements. The assembly results indicated that repeat sequences constituted approximately 20.90% of the genome. Among these repeats, long interspersed nuclear elements (LINEs), short interspersed nuclear elements (SINEs), and long terminal repeats (LTRs) accounted for 4.60%, 0.27%, and 3.93% of the genome, respectively (Fig. 1 and Table 4).

**Table 4:** Statistics of repetitive sequence annotation result.

Type	Length (bp)	% in genome
DNA	61,092,258	9.655
LINE	29,092,985	4.598
SINE	1,681,759	0.266
LTR	24,873,150	3.931
Other	6,780	0.001
Unknown	39,250,152	6.203
Total	132,257,294	20.902

### Gene prediction and functional annotation

We performed gene annotation on the assembled genome, encompassing both structural and functional annotation. Before annotating gene sequences, we masked the observed repetitive sequences. We employed *de novo*, homologue-based, and transcriptomic approaches to predict the location and structure of genes. Subsequently, functional annotation was conducted to unveil the biological roles of these coding genes within the Chinese sea bass genome.

**Fig. 3:**
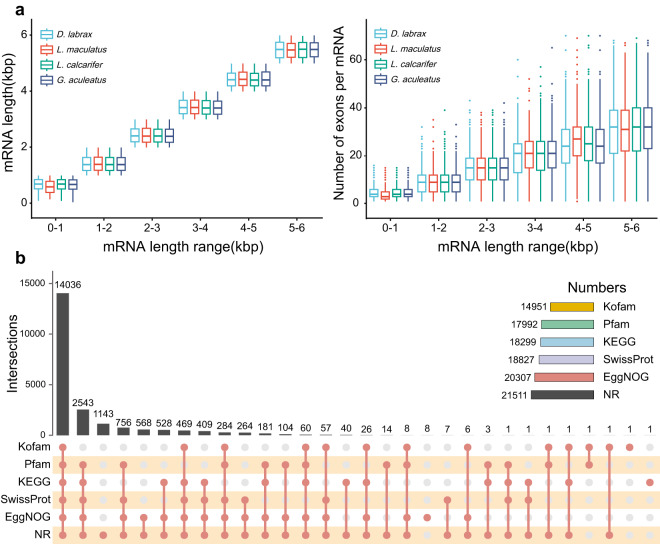
Comparison map of gene sets among closely related species and an UpSet diagram of functional annotation of the Chinese sea bass genome. (**a**) The distribution of mRNA length and the number of exons in each mRNA were compared between gene sets of closely related species using 1 kbp mRNA length as a window. (**b**) Gene function annotation was used to generate a statistical UpSet diagram using 5 public databases: Kofam, Pfam, KEGG, SwissProt, EggNOG, and NR.

We obtained RNA sequencing data from 14 samples of muscle, testis, liver, gill, stomach, spleen, and brain tissues from the NCBI database. These datasets were subsequently aligned to the genome assembly using HISAT2^28^ (v2.1.0) and assembled using StringTie^29^ (v2.1.4). *De novo* prediction of the gene structure within the genome was performed by employing two established fish models, namely, zebrafish and sea lamprey, with the use of the AUGUSTUS^26^ (v3.3.0) gene prediction tool. For homology-based prediction, we utilized Miniport^30^ (v0.11) to conduct a comparative analysis of the protein sequences from 12 closely related species, including *Dicentrarchus labrax*, *Branchiostoma belcheri*, *Gasterosteus aculeatus*, *Cynoglossus semilaevis*, *Lates calcarifer*, *Oreochromis niloticus*, *Danio rerio*, *Oryzias latipes*, *Oryzias melastigma*, *Salmo salar*, *Tetraodon nigroviridis*, *and Takifugu rubripes*. The protein sequences were downloaded from the NCBI database and compared to the genome to infer gene structure according to homology-based evidence. To synthesize the findings obtained from the three methods, we employed EvidenceModeler^31^ (v1.1.1). This powerful tool facilitated the amalgamation and integration of the gene predictions, resulting in the definitive identification of 22,014 protein-coding genes. Gene sets were downloaded from the NCBI database for three species closely related to *L. maculatus*, namely, *D. labrax*, *L. calcarifer*, and *G. aculeatus*. mRNA length distribution and the number of exons in each mRNA was compared among the different gene sets using various length windows (Fig. 3a). The analysis revealed that the statistical characteristics of the gene elements of closely related species exhibited a similar distribution.

After gene prediction, the finalized gene sets derived from the preceding methods underwent functional annotation through matching with a variety of databases. In particular, functional annotation of the inferred genes for *L. maculatus* was performed using diamond^32^ (v2.1.6) against the SwissProt^33^, KEGG^34^, EggNOG^35^, Pfam^36^, NR^37^, and Kofam^38^ databases with an e-value cut-off of 1e-5. Finally, 21,522 genes were annotated, which accounted for 97.77% of all inferred genes of *L. maculatus* (Fig. 3b and Table 5).

**Table 5:** Statistics of functional annotation result.

Type	Number	Percentage (%)
NR	21,511	97.715
EggNOG	20,307	92.246
SwissProt	18,827	85.523
KEGG	18,299	83.124
Pfam	17,992	81.730
Kofam	14,951	67.916
Total	21,522	97.765

## Data Records

The genome assembly data can be accessed at GenBank using the accession number JAUTWU000000000^39^.

The raw sequencing data have been deposited into the CNGB Sequence Archive (CNSA) with the accession number CNP0004610^40^ and Genome Sequence Archive (GSA) in NGDC under the accession number CRA014443^41^.

The genome annotation files, gene CDS, and protein data have been submitted to Figshare^42^.

## Technical Validation

To assess the completeness of the *L. maculatus* genome assembly, we utilized BUSCO^43^ (v5.4.7) with the Actinopterygii database (actinopterygii_odb10) to identify conserved single-copy genes in the assembly. Of the 3,640 conserved genes searched, an impressive 97.9% were identified as complete, indicating a high level of gene content preservation. Among these, 97.2% were both complete and present as single-copy genes, further emphasizing the quality of the assembly. Additionally, only 0.2% were fragmented, and 1.9% were missing from the assembly (Table 6). To ensure the quality and accuracy of the Chinese sea bass assembly, we employed a two-step validation process. First, the assembly quality value (QV) was quantified using Merqury^44^ (v1.4), resulting in a QV score of 54.25, reflecting a high-quality assembly. Subsequently, we aligned the raw sequencing data to the assembly using minimap2^22^ (v2.15). For PacBio HiFi and ONT ultralong sequencing, this alignment approach achieved mapping rates of 99.93% and 99.99%, respectively.

**Table 6:** BUSCO assessment result.

Type	Number	Percentage (%)
Complete BUSCOs	3,564	97.9
Complete and single-copy BUSCOs	3,538	97.2
Complete and duplicate BUSCOs	26	0.7
Fragmented BUSCOs	7	0.2
Missing BUSCOs	69	1.9
Total BUSCO groups searched	3,640	100

## Data Availability

No custom software code was written for this research. All bioinformatics tools and pipelines were executed as per the manual and protocols provided by their respective software developers. The software versions used, along with their corresponding parameters, have been thoroughly described in the Methods section.
